# Discontinuation of hypnotics during cognitive behavioural therapy for insomnia

**DOI:** 10.1186/1471-244X-8-80

**Published:** 2008-09-18

**Authors:** Lucie Zavesicka, Martin Brunovsky, Milos Matousek, Peter Sos

**Affiliations:** 1 Prague Psychiatric Center, Ustavni 91, 18103 Prague 8, Czech Republic; 2 3rd Faculty of Medicine, Charles University, 100 00 Prague 10, Czech Republic

## Abstract

**Background:**

In practical sleep medicine, therapists face the question of whether or not to discontinue the ongoing use of hypnotics in patients, as well as the possible effects of discontinuation. The aim of this study was to evaluate the effects of discontinuing third-generation hypnotics on the results of cognitive-behavioural therapy (CBT) for primary insomnia in patients after long-term abuse.

**Methods:**

Twenty-eight outpatients were treated by CBT for 8 weeks. The treatment outcome was estimated by means of differences among subjective clinical scales and polysomnography variables assessed before and after the treatment period. The therapeutic effect in a subgroup of 15 patients who had previously received hypnotics and were successively withdrawn during weeks 2–6 was compared to the effect achieved in patients who had not used hypnotics before CBT.

**Results:**

There were no significant differences in baseline subjective and objective sleep characteristics between the hypnotic abusers and non-abusers. According to clinical scales and most polysomnographic measures, CBT was highly effective in both groups of subjects; it produced the greatest changes in total sleep time, REM sleep and sleep efficiency. Unexpectedly, discontinuation of hypnotics, as a factor in the analysis, was followed by an additional improvement of sleep efficiency and wake after sleep onset parameters.

**Conclusion:**

Our study confirmed the efficacy of CBT in both hypnotic-abusing and non-abusing patients with chronic insomnia. The results of this study suggest that tapered withdrawal of third-generation hypnotics during CBT therapy for chronic insomnia could be associated with improvement rather than worsening of sleep continuity.

## Background

Many patients suffering from chronic insomnia are prescribed various types of hypnotics, particularly if treated by non-psychiatrists. Thus, there is an increasing group of people in the common population who subscribe to daily, and often long-term, use of hypnotics. In many patients, insomnia continues despite increases in daily hypnotic dose. As a result, specialists are confronted with the difficult task of improving patients' insomnia while concurrently abolishing hypnotic abuse. Some therapists select hypnotics with lower risk of tolerance and dependence. Among these drugs, third-generation hypnotics have become increasingly popular [1-3]. However, it is commonly agreed that psychological and behavioural interventions represent the most effective treatment option for the management of persistent insomnia, as reviewed by Morin et al. [4]. Cognitive-behavioural therapy (CBT) is the mainstay of nonpharmacologic treatments for insomnia, with efficacy that lasts beyond the duration of treatment [5]. The therapist then must decide whether or not to discontinue the administration of hypnotics during CBT. Discontinuation can be particularly difficult in patients of higher age [6]. Although the benzodiazepine-type hypnotics are associated with a higher risk of abuse, the discontinuation of drug intake seems to be facilitated by CBT [7]. On the other hand, some authors may choose to continue the administration of hypnotics in order to facilitate the therapeutic effect of CBT [8-10].

Therefore, there is no commonly agreed method of treatment for patients who seek CBT after several years of daily use of hypnotics. The uncertainty particularly concerns the long-term use of third-generation hypnotics with a low risk of abuse. The intention of the present study was to elucidate some of the questions that confront the therapist. Thus, the aim was to contribute to the answers to the following questions:

1. Does treatment outcome with CBT for insomnia vary between subjects with respect to hypnotic abuse?

2. Do baseline sleep characteristics, including sleep continuity and sleep architecture differ significantly with respect to the abuse of third-generation hypnotic therapies?

3. What is the CBT treatment outcome for insomnia in patients after discontinuation of long-term hypnotic medication, compared with treatment outcome in patients who have used no hypnotics at all?

To clarify these questions, two subgroups of patients who had or had not abused third-generation hypnotics for a long time before CBT for insomnia, were examined. In the subgroup of patients who had abused hypnotics, medication was discontinued during the first part of the CBT period. This investigation was not a comparative study, and should be considered as a clinical case series that had two primary factors of interest: 1) Group (hypnotic abusers [n = 15] vs. subjects without hypnotic abuse [n = 13]), and 2) Time (baseline vs. end of treatment). The effect of CBT was estimated according to differences among clinical scales and sleep indicators obtained by means of polysomnography recordings before and after the CBT treatment period.

## Methods

### Patients

The study was based on 28 consecutive outpatients (19 females and 9 males) aged 25 to 74 years (mean 44.35 years, s.d. 13.87) who had been diagnosed with primary insomnia (F 51.0) according to ICD-10 [11]. The mean duration of the disorder was 4.75 years (s.d. 1.60). Patients with psychotic disorder, bipolar disorder, major depression, personality disorders, chronic alcoholism, dementia or other organic brain disorders were not included in the study. All patients were in good physical health. According to the polysomnography examination conducted as part of the study, no signs of sleep-disordered breathing could be found in any of the patients. Among the patients, 15 had used zolpidem for more then one year in doses of 15–20 mg daily (n = 12), 30–40 mg daily (n = 2), and 70 mg daily (n = 1). The patients did not use any another drug (such as benzodiazepines) for at least two months before beginning the study. The characteristics of this subgroup ("hypnotic abusers"), compared with the subgroup of patients without hypnotics abuse ("hypnotic non-abusers", n = 13) can be found in Table 1.

**Table 1:** Baseline demographics and characteristics, and clinical and comparative polysomnographic data from two subgroups of subjects.

	Hypnotics non-abusing (N = 13)	Hypnotics-abusing (N = 15)	Significance
Age [years]	45.23 (s.d. 14.53)	42.27 (s.d. 13.54)	p = 0.58^a^
Gender [female : male]	9 : 4	10 : 5	p = 0.60^b^
Duration of disorder [insomnia length in months]	48 (IQR 17–204)*	60 (IQR 24–147)*	p = 0.62^c^
Insomnia Severity Index [ISI]	16.54 (s.d. 4.19)	18.67 (s.d. 4.93)	p = 0.23^a^
Epworth Sleepiness Scale [ESS]	12.15 (s.d. 5.83)	11.40 (s.d. 4.88)	p = 0.71^a^
Beck Depression Inventory [BDI]	7.0 (IQR 2–11)*	6.5 (IQR 2.0–9.25)*	p = 0.56^c^
Beck Anxiety Inventory [BAI]	10.31 (s.d. 6.21)	9.87 (s.d. 6.79)	p = 0.86^a^
Hamilton Rating Scale for Depression [HAMD]	7.85 (s.d. 3.36)	5.80 (s.d. 3.38)	p = 0.12^a^
modified Clinical Global Impression Severity [mCGI-S]	10 (IQR 9–11)*	8 (IQR 7–10)*	p = 0.18^c^
Total sleep time TST [min]	344.69 (s.d. 58.14)	292.63 (s.d. 143.06)	p = 0.23^a^
Sleep latency SL [min]	26.0 (IQR 7.15–47.45)*	27.2 (IQR 12.63–45.94)*	p = 0.72^c^
Sleep efficiency SE [%]	75.5 (IQR 63.5–82.0)*	72.0 (IQR 64.0–81.0)*	p = 0.86^c^
Wake after sleep onset WASO [min]	72.42 (s.d. 55.84)	87.37 (s.d. 58.72)	p = 0.50^a^
Stage 1 [min]	36.50 (s.d. 19.74)	31.90 (s.d. 24.50)	p = 0.59^a^
Stage 2 [min]	153.96 (s.d. 82.50)	154.37 (s.d. 40.50)	p = 0.98^a^
Stage 3+4 [min]	105.40 (s.d. 51.00)	120.74 (s.d. 44.50)	**p = 0.04^a^**
REM [min]	55.90 (s.d. 45.00)	76.77 (s.d. 39.00)	**p = 0.04^a^**

* Data are presented as median (interquartile range) due to non-normal data distribution. Characters in bold indicate significance (p < 0.05).

^a ^– One-way ANOVA; ^b ^– Fischer Exact Test; ^c ^– Mann-Whitney U Test;

IQR – interquartile range; s.d. – standard deviation; N.S. – non significant

The Prague Psychiatric Centre Institutional Review Board reviewed and approved this study, and written informed consent to participate in the research was obtained from all subjects. The study was carried out in accordance with the latest version of the Declaration of Helsinki.

### Clinical examination

The clinical examination consisted of a psychiatric interview and physical examination supplemented with a study of sleep quality. All patients kept sleep logs, starting one week before the first polysomnograph. The sleep interviews and the medical examination were conducted by a physician board-certified in psychiatry and sleep medicine. To characterize the insomnia more precisely and to assess therapeutic outcomes, the Insomnia Severity Index (ISI) [12] and Epworth Sleepiness Scale (ESS) [13] were completed prior to and following CBT. The following scales and inventories were also applied, both before and after CBT: the Beck Depression Inventory (BDI) [14,15], the Beck Anxiety Inventory (BAI) [16] and the Hamilton Rating Scale for Anxiety (HAMD) [17]. Besides that, we also completed our modified version of Clinical Global Impression Severity scale (mCGI-S) ranged from 2 (not ill) to 14 (extremely severely ill), which summed together a standard version of CGI [18] rated from 1 (normal, not at all ill) to 7 (among the most extremely ill patients) and a separate scale (with the same range) that evaluated solely clinical global impression of a severity of sleep disturbance and sleep quality. The assessment of our modified Clinical Global Impression Improvement scale (mCGI-I), which evaluate patient's improvement or worsening with the range from 2 (very much improved) to 14 (very much worse), was performed in similar way. Separate baseline scores for each subgroup can be found in Table 1.

### Study design

This investigation was based on the comparison of data obtained from patients who were hypnotic abusers, and patients without abuse potential. The examination and therapy were the same in both subgroups. The study began with an initial one-week observation period during which the patients were asked to fill in their sleep logs. Afterwards, a polysomnographic examination ("adaptive") was done. On the following day, the subjective scores were registered and another polysomnographic examination ("baseline") was performed. CBT (see below) was then applied over the next 8 weeks. Fifteen hypnotics abusing patients were provided with a step-by-step withdrawal schedule, with the goal of eliminating abuse of third-generation hypnotics by the 2^nd ^to 6^th ^week (mostly in the 4^th ^week) of treatment. We used several principles (according to [7]), including 1) setting goals, 2) stabilization with use of a single type of third-generation hypnotic, 3) reduction of the initial dose by roughly 25% per week until the lowest available dose was reached, and 4) introduction of an increasing number of medication-free nights. The initial plan was to decrease medication by 25% at week 1, by 50% at week 2, and by 100% at week 4. The day after the treatment period ended, subjective scores were registered and a third polysomnographic examination ("post-treatment") was conducted.

### Polysomnographic recording

The polysomnography concerned electroencephalogram (EEG) derived from 9 channels (F3-T3, T3-T5, T5-O1, F8-T4, T4-T6, T6-O2, Fz-Cz, T3-Cz, and Cz-T4), electrocardiogram (ECG), naso-oral airflow, chest movements, oxygen saturation, electrooculogram (EOG), and electromyogram (EMG) from the submandibular, thoracic, and anterior tibialis muscles. The time to switch the light off was chosen by the patient. Recording was then started and continued until spontaneous awakening in the morning. The polysomnographic records were scored "blind" by an expert according to Rechtschaffen and Kales' criteria [19].

### Psychotherapy

Psychotherapy consisted of 8 sessions (each lasting 1 hour) of cognitive-behavioural group treatment for chronic insomnia. Each group consisted of 5 patients. The purpose of this treatment was to help the patients to identify and modify their dysfunctional insomnia-related thoughts, beliefs, and behaviour, and to break the recurring cycle of anticipatory anxiety. The major components of CBT were:

(a) sleep education and cognitive restructuring concerning sleep (i.e., recognizing, challenging, and changing distorted or inaccurate attitudes and beliefs about sleep requirements, attributions, effects of sleep loss, and subjective perception of amount of sleep obtained),

(b) information on sleep hygiene (i.e., reducing alcohol, caffeine, and nicotine use; increasing daytime exercise but not within 4 hours of bedtime; establishing a regular wind-down period prior to bedtime; avoiding stimulating mental and physical activities before bedtime; maintaining a quiet, cool, and dark bedroom; etc.),

(c) behaviour therapy, sleep restriction, and stimulus control (employing a regular arising time; limiting time in bed to 1.5 hours beyond the average sleep length, as calculated from weekly sleep diaries, to improve sleep efficiency; using the bedroom for sleep or relaxing activities only; going to bed only when drowsy; and, if not asleep within 20 to 30 minutes, opening eyes and engaging in relaxing activity in bed or another room with no attempt to sleep until drowsy again, with repetition as necessary),

(d) cognitive therapy, work with autonomic negative though, work with behaviour, emotion, thoughts, physical reaction and cognitive restructuring (i.e., recognizing, challenging, and changing distorted negative cognitive appraisals concerning daily stressors), and

(e) progressive relaxation (a set of integrated physiologic changes that are consistent with reductions in sympathetic nervous system activity and that are elicited when an individual engages in a repetitive mental activity – muscular relaxation and breath focusing, while passively ignoring distracting thoughts) [20-22].

Sessions were supplemented with educational and directive reading materials.

### Statistical analysis

To compare the pre- and post-treatment variables, the paired t-test and ANOVA were applied. For data with non-normal statistical distribution, nonparametric statistical tests were used to perform within group (Wilcoxon Sign Rank Test) and between group (Mann-Whitney U Test and Fisher Exact Test) analyses (statistical software SPSS v.12.0). The intention was to examine the changes in sleep variables achieved by therapy. The influence of hypnotics' discontinuation was investigated, as a possible factor regarding various sleep parameters by General Linear Model completed by post-hoc t-test. Differences between the pre- and post-treatment values were used as input data for investigating changes during therapy.

## Results

### Effects of hypnotic abuse on subjective and objective sleep variables

At the beginning of the treatment period, both subgroups of patients were examined using traditional whole-night sleep polysomnography. In this part of the study, pre-treatment data obtained from the hypnotic abusing patients were compared to data obtained from the non-abusing patients. The mean values of the sleep indicators (subjective and objective clinical scales, sleep continuity, and sleep architecture) for both subgroups are displayed separately in Table 1. While sleep was clearly disturbed in all patients, there were no significant differences in baseline subjective and objective clinical scales values between hypnotic abusers and non-abusing patients. Among polysomnographic parameters the proportion of slow wave sleep of stages 3 and 4 was relatively increased in those patients abusing third-generation hypnotics. Similarly, the proportion of REM sleep was higher in hypnotic-abusing patients than in non-abusing ones.

### Effects of CBT on clinical scales and sleep variables

The differences between pre- and post-treatment sleep variables served as indicators of treatment outcome. In this part of the study, the therapeutic result was evaluated in all 28 patients, with both subgroups combined. The baseline data obtained by subjective clinical scales and polysomnography before therapy are displayed in the second column of Table 2. The table shows the means/medians and standard deviations/interquartile ranges as registered in the entire group, including both hypnotic-abusing and non-abusing patients. Sleep disturbance was reflected, for example, in the measure of sleep efficiency, with a mean value of 77.58% (± 19.28). The other polygraphic variables, obtained before therapy, are also revealed in the second column ("pre-treatment"). The mean/median values of the same clinical scales and sleep variables, obtained after CBT are displayed in the third column ("post-treatment"). A statistical comparison between the pre- and post-treatment data reveals a significant improvement in almost all sleep parameters after therapy. The exceptions was the amount of sleeping time spent in sleep 2 and sleep 1 stages, which were somewhat lower after CBT, but the difference was not statistically significant.

**Table 2:** Comparison of clinical symptom scales and sleep parameters before and after CBT, n = 28.

	Pre-treatment	Post-treatment	Significance
Insomnia Severity Index [ISI]	17.68 (s.d. 4.49)	10.89 (s.d. 5.40)	**p < 0.001^a^**
Epworth Sleepiness Scale [ESS]	11.75 (s.d. 5.25)	8.64 (s.d. 5.07)	**p = 0.03^a^**
Beck Depression Inventory [BDI]	7.0 (IQR 2.0–10.0)*	2.0 (IQR 0–6.0)*	**p < 0.001^b^**
Beck Anxiety Inventory [BAI]	10.07 (s.d. 6.41)	5.39 (s.d. 4.43)	**p = 0.002^a^**
Hamilton Rating Scale for Depression [HAMD]	6.5 (IQR 3.0–10.5)*	1.0 (IQR 0–1.5)*	**p < 0.001^b^**
modified Clinical Global Impression [mCGI] *(mCGI-Severity for pre-treatment and mCGI-Impression for post-treatment conditions)*	9.0 (IQR 8.0–10.0)*	4.0 (IQR 2.0–4.5)*	**p < 0.001^b^**
Total sleep time TST [min]	361.80 (s.d.113.20)	454.12 (s.d.59.37)	**p = 0.01^a^**
Sleep latency SL [min]	26.0 (IQR 11.98–46.10)*	7.25 (IQR 3.83–13.0)*	**p = 0.004^b^**
Sleep efficiency SE [%]	77.58 (s.d. 19.28)	89.60 (s.d. 8.43)	**p = 0.004^a^**
Wake after sleep onset WASO [min]	80.42 (s.d. 56.85)	48.27 (s.d. 37.17)	**p = 0.02^a^**
Stage 1 [min]	28.5 (IQR 22.0–41.75)*	22.75 (IQR 16.5–37.0)*	p = 0.29^b^
Stage 2 [min]	154.48 (s.d. 51.43)	149.12 (s.d. 55.72)	p = 0.72^a^
Stage 3+4 [min]	112.52 (s.d. 80.62)	175.09 (s.d. 94.09)	**p = 0.01^a^**
REM [min]	65.59 (s.d. 43.49)	107.91 (s.d. 49.12)	**p = 0.001^a^**

In most cases data are expressed as the mean (standard deviation). Characters in bold indicate significance (p < 0.05).

* Data are presented as median (interquartile range) due to non-normal data distribution

^a ^– Paired-Samples T test; ^b ^– Wilcoxon Sign Rank Test;

IQR – interquartile range; s.d. – standard deviation; N.S. – non significant

### Effects of hypnotic discontinuation on sleep variables

The differences between pre- and post-treatment sleep variables also served as indicators of treatment outcome. The intention of this part of the study was to compare treatment outcomes achieved in the hypnotics-abusing patients to outcomes achieved in non-abusing patients. Thus, statistical analysis should evaluate whether the treatment outcome (i.e., difference between post- and pre-treatment variables) was influenced by past hypnotic abuse and withdrawal of hypnotics during the treatment period. Improvement could be seen in all patients in both subgroups, and possible additional effects of the withdrawal of hypnotic were investigated. Sleep efficiency (SE) and wake after sleep onset (WASO) improved significantly after the discontinuation of third-generation hypnotics (Table 3).

**Table 3:** Changes in clinical symptom scales and sleep parameters after CBT presented for the separate subgroups.

	Hypnotics non-abusing (N = 13)	Hypnotics abusing (N = 15)	Significance
Insomnia Severity Index [ISI]	-5.7 (s.d. 4.93)	-6.9 (s.d. 3.97)	p = 0.48^a^
Epworth Sleepiness Scale [ESS]	-2.00 (IQR -4.0 – 0)*	-1.5 (IQR -2.5 – 0.25)*	p = 0.43^b^
Beck Depression Inventory [BDI]	-4.33 (s.d. 4.53)	-3.50 (s.d. 3.94)	p = 0.61^a^
Beck Anxiety Inventory [BAI]	-5.00 (s.d. 4.60)	-4.71 (s.d. 5.77)	p = 0.88^a^
Hamilton Rating Scale for Depression [HAMD]	-4.0 (IQR -9.0 – -2.0)*	-3.50 (IQR -9.75 – -2.75)*	p = 0.98^b^
modified Clinical Global Impression Improvement [mCGI-I]	4.0 (IQR 3.0–5.0)*	3.0 (IQR 2.0–4.0)*	p = 0.16^b^
Total sleep time TST [min]	84.60 (s.d. 101.36)	83.28 (s.d. 137.99)	p = 0.98^a^
Sleep latency SL [min]	-12.25 (IQR -25.0 – 2.0)*	-17.00 (IQR -31.68 – -5.75)*	p = 0.48^b^
Sleep efficiency SE (%)	6.16 (s.d. 6.12)	19.95 (s.d. 25.0)	**p = 0.02^a^**
Wake after sleep onset WASO (min)	-25.81 (s.d. 28.69)	-36.86 (s.d. 71.94)	**p = 0.003^a^**
Stage 1 [min]	-4.20 (s.d. 21.17)	-2.64 (s.d. 26.11)	p = 0.87^a^
Stage 2 [min]	-3.43 (s.d. 56.79)	-4.88 (s.d. 49.25)	p = 0.94^a^
Stage 3+4 [min]	68.34 (s.d. 98.00)	61.90 (s.d. 86.46)	p = 0.86^a^
REM [min]	45.88 (s.d. 69.41)	36.23 (s.d. 52.13)	p = 0.68^a^

In most cases change is expressed as the mean difference (standard deviation) between values obtained after and before therapy. Characters in bold indicate significance (p < 0.05).

* Data are presented as median (interquartile range) due to non-normal data distribution

^a ^– One-way ANOVA; ^b ^– Mann-Whitney U Test;

IQR – interquartile range; s.d. – standard deviation; N.S. – non significant

### Effects of discontinuation of hypnotics on clinical variables

The above described results were obtained by means of polysomnography. It was of interest to determine whether changes in polysomnographic variables were paralleled by changes in clinical variables. Parallel changes would suggest a possible relationship between improvement in sleep variables and improvement in clinical scores. A parallel relationship was found between diminished anxiety (Beck Anxiety Inventory) and a decrease in WASO. A detailed analysis of this result, as displayed in Table 4, revealed that the decrease in WASO was actually influenced by a combination of two factors: decrease of anxiety, and discontinuation of hypnotics.

**Table 4:** Effect of hypnotic discontinuation and reduced anxiety as possible factors reducing wake after sleep onset. Dependent variable: WASO (wake after sleep onset).

Source	Sum of Squares	df	Mean Square	F-value	Significance
Corrected Model	25643.428	6	4273.905	2.425	**p = 0.04**
Intercept	6782.891	1	6782.891	3.848	p = 0.06
Hypnotic withdrawn * BAI	9464.062	1	9464.062	5.369	**p = 0.03**
Hypnotics withdrawn	19912.822	1	19912.822	11.297	**p = 0.003**
BAI	8910.463	1	8910.463	5.055	**p = 0.04**
Error	37015.599	21	1762.648		
Total	91619.750	28			
Corrected Total	62659.027	27			

df – degree of freedom; BAI – Beck Anxiety Inventory;

Characters in bold indicate significance (p < 0.05).

## Discussion

Many patients suffering from chronic insomnia participate in long-term daily use of hypnotics. Those who are inadequately treated have a tendency to increase their daily dose in an effort to combat their continuing insomnia. Such patients are clearly at risk for hypnotics abuse and dependence. Application of CBT for insomnia is the best way to avoid such developments and to ensure proper therapy in these patients. Therapists are less certain, however, what to do regarding the ongoing hypnotic regimen during CBT. Discontinuation of hypnotic administration is not always possible, because both the patient and the therapist may be concerned about worsening insomnia. There is a fear that abrupt withdrawal of hypnotics could result in worse therapeutic outcomes. However, practical experience with discontinuation of hypnotics does not justify this concern. There are reports on long-term outcomes of discontinuing hypnotics, particularly benzodiazepines [7,10].

On the other hand, experience concerning other types of hypnotics is rather limited. Therefore, a study aiming to contribute further observations regarding therapeutic outcomes after discontinuing hypnotics has been initiated. In contrast to previous studies, this study was focused on third-generation hypnotics [8,10,23]. Although the risks of abuse and dependence seem to be lower in third-generation hypnotics than in other hypnotic drug classes, withdrawal of third-generation hypnotics is also often desirable. The study should elucidate which changes in sleep characteristics are caused by a long-term intake of the drugs and, in particular, what the therapeutic outcome would be after their discontinuation.

In this study, two subgroups of patients, with and without a history of hypnotic abuse, were examined. To shed light on the effect of a long-term hypnotics regimen, pre-treatment sleep indicators were considered. In hypnotics-abusing patients a relative increase in sleep stage 3, sleep stage 4, and REM sleep could be demonstrated in comparison with the hypnotic non-abusing subgroup. Both subgroups of patients were then treated with CBT while the long-term hypnotic regimen was gradually discontinued. Afterwards, the therapeutic outcomes were evaluated, using the differences between pre- and post-treatment sleep variables as indicators. Sleep was significantly improved in both subgroups after CBT. The only exception was the proportion of sleep stages 1 and 2, in which statistical significance could not be confirmed. As a next step, the possible effects of discontinuing hypnotics were investigated. Statistical analysis revealed an additional improvement of sleep efficiency and WASO in the abusing group after therapy.

One possible objection is that several factors influencing sleep in parallel could not be studied separately because it was necessary to respect the patients' interests to obtain efficient therapy by means of CBT [9]. These drawbacks are certainly compensated for by using an objective method to follow the quality of sleep as well as changes in sleep quality. Many studies are based on the subjective reports of patients, even though, as shown in our previous study, subjective data may be less reliable than expected [24]. Researchers are well aware of the fact that evaluation of subjective variables is to be expected in a study of this type. However, considering that the unreliability of subjective sleep data variables has been previously demonstrated, it would certainly be improper to continue to use them in future studies of this type. To avoid any confusion due to the use of insufficient data, it was decided to base findings and conclusions on objective variables obtained by means of polysomnographic examination.

Several questions remain unanswered, and a new study, conducted independently and with a more extensive study population, will be necessary to confirm these findings. However, enrolment of a large patient population, particularly of patients without any previous medication history, is a difficult task. Of course, results for which the statistical significance was confirmed can hardly be explained by insufficient statistical power. On the contrary, it is only plausible to expect more pronounced changes and higher significance with a larger patient population. Another possible objection concerns therapeutic outcomes. This study does not provide information regarding the durability of therapeutic effects, and a follow-up study will therefore be necessary. Furthermore, considering the practical consequences of the study, it should be emphasized that these findings concern third-generation hypnotics only. Possible interference with other medications was avoided in this study. Thus, the experience gained from this study is not necessarily applicable to all types of patients or hypnotic medications.

## Conclusion

In summary, the present findings extend those from previous studies in documenting the **e**fficacy of CBT in treating chronic primary insomnia. Summarizing all the positive and negative points of our study, it seems indisputable that the discontinuation of long-term daily hypnotic regimens can be done without any hesitation in patients with primary insomnia. The results of the study suggest that withdrawal of third-generation hypnotics can improve rather than worsen the results of CBT in treating chronic insomnia.

## Competing interests

The authors declare that they have no competing interests.

## Authors' contributions

LZ was the principal investigator of the study. LZ, MB, and MM wrote the manuscript. All authors contributed to the manuscript. MM made substantial contributions to data analysis and interpretation, as well as manuscript preparation. LZ and MB designed sleep protocols, trained sleep technicians, and supervised sleep studies. LZ and PS carried out CBT treatment and supervised medication tapering, conduct of the clinical study itself, and data collection. MM supervised and participated with great impact at all stages of manuscript preparation, and conducted the statistical analyses together with PS. All authors read and approved the final manuscript.

## Pre-publication history

The pre-publication history for this paper can be accessed here:


